# Impact of Virtual Reality Alone and in Combination with Conventional Therapy on Balance in Parkinson’s Disease: A Systematic Review with a Meta-Analysis of Randomized Controlled Trials

**DOI:** 10.3390/medicina61030524

**Published:** 2025-03-17

**Authors:** Giorgio De Natale, Erda Qorri, Jasemin Todri, Orges Lena

**Affiliations:** 1 GDN Fisioterapia Avanzata, Via Bellini 55, 95030 Sant’Agata Li Battiati, Italy; gdenatale@alu.ucam.edu; 2 ITEM-Innovation in Manual and Physical Therapies Research Group, Physiotherapy Department, UCAM Universidad Católica de Murcia, 30107 Murcia, Spain; lorges@ucam.edu; 3 Department of Dentistry, Faculty of Medical Sciences, Albanian University, 1001 Tirana, Albania; e.qorri@albanianuniversity.edu.al

**Keywords:** Parkinson’s disease, virtual reality, traditional exercise, balance, fall risk

## Abstract

*Background and Objectives*: Virtual reality (VR)-based interventions provide immersive and interactive environments that can enhance motor learning and deliver real-time feedback, offering potential advantages over conventional therapies. This systematic review evaluated the impact of non-immersive and immersive VR exergaming interventions versus conventional therapy on balance in Parkinson’s disease (PD) through a detailed analysis of randomized controlled trials (RCTs). *Materials and Methods*: A comprehensive search was conducted across the PubMed, Lilacs, IBECS, CENTRAL, Web of Science (WOS), EBSCOHost, and SciELO databases. Article selection and duplicate removal were managed using Rayyan QCRI. The quality of the evidence was assessed using the GRADE system. *Results*: From an initial screening of 100 studies, 58 underwent title and abstract screening. After full-text evaluation, 11 RCTs met the inclusion criteria, involving 518 participants with PD (average age: 67.3 years; 67.95% men). The balance outcomes were primarily measured using the Berg balance scale (BBS), employed in most studies (n = 9). The pooled analysis demonstrated a significant improvement in the balance scores for the experimental groups compared to the controls, with a standardized mean difference (SMD) of 0.58 [95% CI: 0.07, 1.09, *p* = 0.03]. However, the heterogeneity was substantial (I^2^ = 77%). The analysis of the six-minute walking test (6 MWT), as another outcome of four articles, revealed a mean difference of 32.99 m [95% CI: −8.02, 74.00], but the effect was not statistically significant (*p* = 0.11). The heterogeneity for this outcome was moderate (I^2^ = 41%), indicating some variability across studies. Alternative tools, such as the Tinetti Performance-Oriented Mobility Assessment (POMA) scale, the UPDRS III, and the sensory organization test (SOT), were also evaluated where possible. *Conclusions*: VR-based interventions offer promise for improving balance in Parkinson’s disease, enhancing rehabilitation engagement. Their integration into clinical practice could complement conventional therapy. However, further research is needed to optimize protocols, standardize parameters, and maximize their impact on mobility, independence, and quality of life.

## 1. Introduction

Parkinson’s disease (PD) is a progressive neurodegenerative disorder that significantly impairs motor functions, particularly balance, leading to an increased risk of falls and diminished quality of life [1]. Traditional rehabilitation methods have aimed to address these balance deficits; however, recent advancements have introduced virtual reality (VR) as a promising therapeutic modality [2]. VR-based interventions offer immersive and interactive environments that can enhance motor learning and provide real-time feedback, potentially surpassing the benefits of conventional therapies [3]. Several systematic reviews and meta-analyses have evaluated the efficacy of VR interventions on balance in individuals with PD [4,5,6]. For instance, a study by Dockx et al. (2016) analyzed randomized controlled trials comparing VR training to traditional balance training, concluding that VR-based rehabilitation is a promising intervention for improving the balance function in patients with PD [7]. Similarly, a systematic review and meta-analysis by Wang et al. (2021) found that VR rehabilitation led to more significant improvements in balance compared to traditional exercises, suggesting its utility as an adjunctive method in PD rehabilitation [8]. VR-based interventions leverage immersive, interactive environments that simulate real-life scenarios, providing a safe and controlled space for balance training [9]. These interventions are designed to enhance motor learning, adaptability, and neuroplasticity through repetitive, task-specific exercises with real-time feedback. Furthermore, VR systems can be tailored to the specific needs and abilities of individuals with PD, enhancing motivation and engagement compared to conventional therapies [10]. Similarly, Bekkers et al. (2020) found that combining virtual mobility exercises with treadmill training enhanced gait stability and reduced freezing episodes in early-to-moderate PD [11]. Beyond physical benefits, VR also addresses the psychosocial dimensions of PD. The immersive and interactive nature of VR can reduce feelings of isolation by enabling participation in virtual group activities or environments that mimic social interactions [12]. This holistic approach to rehabilitation is particularly valuable, as PD often entails cognitive and emotional challenges alongside motor impairments.

Despite the promising evidence, several gaps remain in the literature. Variability in study designs, VR platforms, intervention durations, and outcome measures poses challenges in drawing definitive conclusions. Furthermore, the long-term sustainability of VR-induced improvements in balance and its impact on fall rates remain underexplored.

VR-induced improvements in PD are defined through measurable clinical and functional outcomes. These include enhanced gait and balance, improved motor function, and cognitive benefits (evaluated through neuropsychological tests). Additionally, reductions in the freezing of gait (FoG) are quantified through patient reports and objective analysis. Quality of life and psychological well-being are assessed using standardized questionnaires.

Referring to the mentioned gaps, a new systematic review with a meta-analysis comparing virtual reality (VR) and traditional exercise in Parkinson’s disease (PD) is essential due to the rapid advancements in VR technology, inconsistencies in previous findings, and the need for a comprehensive analysis of motor and non-motor outcomes. It allows for the identification of patient subgroups that benefit the most, addresses methodological limitations in prior studies, and provides updated evidence to guide clinical practice. Additionally, it supports future research by highlighting gaps and informing policy decisions, ultimately contributing to the development of standardized rehabilitation protocols for PD management.

For this reason, this systematic review aims to evaluate the recent impact of VR interventions on balance in PD through a comprehensive analysis of randomized controlled trials (RCTs). Specifically, it seeks to accomplish the following:

1. To assess the additional benefits of integrating VR with conventional therapy on balance in PD;
2. To compare the efficacy of VR-based interventions versus conventional therapies on balance-related outcomes.

By addressing these objectives, this review aims to inform evidence-based clinical practice and guide the development of optimized rehabilitation strategies for individuals with PD.

## 2. Methods

### 2.1. Design

A systematic review with a meta-analysis was conducted in accordance with the Preferred Reporting Items for Systematic Reviews and Meta-Analyses (PRISMA) guidelines [13]. This systematic review was registered on the International Prospective Register of Systematic Reviews (PROSPERO) database (CRD42025642047) and on Open Science Framework (https://osf.io/dkj5w, accessed on 31 January 2025) with the following registration DOI: https://doi.org/10.17605/OSF.IO/DKJ5W.

### 2.2. Consulted Documentary Sources

For this systematic review, the following digital health databases were consulted through a cut-off time from January 2010 to December 2024: “PubMed” through its “National Library of Medicine” platform; Lilacs and IBECS via their Virtual Health Library platform; EBSCOHost, CENTRAL through the Cochrane Library platform; the WOS Core Collection using “Web of Science”; and SciELO.

### 2.3. Research Strategy

Two searches were performed using a combination of terms following the PICOS strategy [14] from the English MeSH thesaurus. The first search focused on terms such as “Parkinsonian Disorders”, “Exergaming”, and “Physical Therapy Modalities”. The second search included the terms “Parkinson Disease” [MeSH] AND “Virtual Reality Exposure Therapy” [MeSH] AND “Physical Therapy Modalities” [MeSH]. All terms in the searches were required to appear in the title, abstract, keywords, thesauruses, MeSH subheadings, study type, author, and institution. The terms were combined using the Boolean operator AND. The search strategies specific to each database are outlined in the Annex Section.

### 2.4. Eligibility Criteria

Randomized controlled clinical trials published in national and international journals up to first December 2024 were included, in which the effectiveness of virtual reality treatment versus conventional treatment was individually assessed or compared in patients with Parkinson’s disease. Studies where both therapies were evaluated together were also included. Additionally, studies had to measure improvement in at least one of the following variables: gait and distance achieved, static and dynamic balance, fall risk, cognitive functions, upper limb motor skills, and quality of life, as these were the focus of this review.

Studies were excluded if they analyzed the effect of VR treatment combined with therapies other than conventional ones, which could obscure the results. Studies that combined different types of neurological diseases other than Parkinson’s and those where no intervention was implemented in the control group were also excluded.

### 2.5. Study Selection Process

The management of the selection process for the articles identified through the bibliographic search and the removal of duplicate studies in databases was carried out using the Rayyan QCRI manager, a software tool used for screening articles [15]. The article selection process was performed by two independent researchers in two distinct phases. The first phase began once the total number of studies was identified; an initial screening was conducted by reading the titles and abstracts. Based on the selected articles, the second phase involved reading the full texts to verify whether they met the inclusion and exclusion criteria.

### 2.6. Data Extraction

Two researchers independently extracted the data, and a third evaluator subsequently verified all the extracted variables. The PICOS strategy was used for this extraction, allowing for the identification of the essential data needed to develop the entire research. Additionally, data were extracted regarding the participants’ characteristics (sex, sample size, age, severity of the condition), the intervention characteristics (frequency, intensity, session duration), the general characteristics of the control groups, and variables related to balance:

Population (3): adult, aged 65+, Parkinson’s disease;
Intervention (1): virtual reality;
Comparison (1): conventional physiotherapy;
Outcomes (5): traditional exercise, Berg balance scale, sensory organization test score, six-minute walking test, Tinetti Performance-Oriented Mobility Assessment.

### 2.7. Risk-of-Bias Assessment Tool

The risk-of-bias assessment for the included studies was carried out individually for each author. The tool used for this assessment was the one proposed by the Cochrane Handbook for Systematic Reviews of Interventions [16]. This tool includes six specific domains, with a score for each domain that can be rated as high, medium, or low risk of bias. The domains assessed using this tool are as follows: sequence generation (selection bias), sequence concealment (selection bias), blinding of participants and personnel (performance bias), blinding of outcome assessors (detection bias), incomplete outcome data (attrition bias), selective reporting of outcomes (reporting bias), and other biases.

### 2.8. Quality of Evidence

The quality level of the scientific evidence was assessed using the Grading of Recommendations, Assessment, Development, and Evaluation (GRADE) system [17]. This system assesses the quality of the evidence by measuring the level of confidence users have in the reported effect of the evaluated element. The GRADE assessment of the evidence quality includes factors such as the risk of bias in the studies, inconsistency, imprecision, publication bias, indirect results, and other factors that may influence the quality of the evidence. To facilitate the consultation of this information, summary tables of findings are developed.

### 2.9. Treatment Effect Analysis

Two review authors independently classified the outcome measures based on the domain assessed. When a study reported multiple outcome measures for the same domain, the most frequently used measure across studies was selected. Risk ratios (RRs) with 95% confidence intervals (CIs) were calculated for dichotomous outcomes, while mean differences (MDs) or standardized mean differences (SMDs) were used for continuous outcomes, as appropriate. All analyses were conducted using Cochrane’s Review Manager 5.4 software. The statistical significance threshold was established at *p* < 0.05. Heterogeneity among studies was evaluated visually using forest plots, as well as through the chi-square test and the I^2^ statistic. The I^2^ values were interpreted as follows: not important (<40%), moderate (30–60%), substantial (50–90%), and considerable (75–100%), with caution advised in their interpretation.

### 2.10. Data Synthesis

A random-effect model meta-analysis was conducted whenever feasible. If a meta-analysis was not possible due to significant variability among studies or the identification of only a single study, a narrative review was provided instead.

When feasible, subgroup analyses were performed to assess whether the outcomes varied based on factors such as age, disease duration, disease severity, intervention frequency (sessions per week), intervention intensity (total hours of intervention), and intervention type (a traditional rehabilitation program versus a commercial gaming console).

## 3. Results

### 3.1. Study Selection and Identification Process

**Figure 1:**
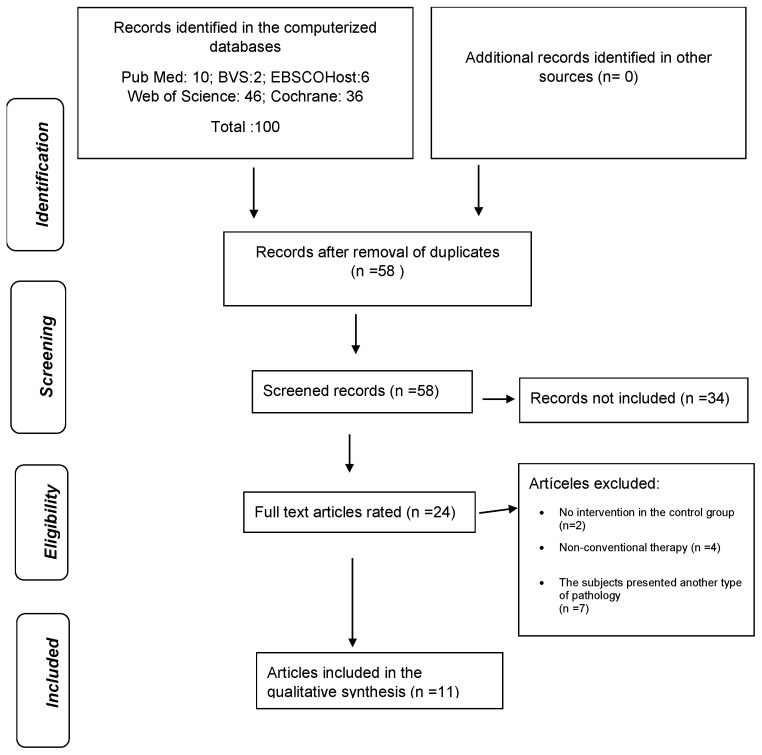
Flowchart of the study selection process.

A total of 100 research articles were retrieved from the consulted databases. After removing duplicates, titles, and abstracts, 58 studies were evaluated. Among them, 24 trials met the inclusion criteria. After reviewing the full texts, seven studies were excluded for failing to meet the specific criteria and for including irrelevant pathologies. Four additional studies were excluded for combining VR treatment with non-conventional therapies, and two were excluded due to the absence of an intervention in the control group. Finally, 11 trials were included in this systematic review (Figure 1).

### 3.2. General Characteristics of the Included Studies

This review included 11 randomized controlled clinical trials published between 2014 and 2024, with 2019 having the highest number of published studies. Three of the included studies were conducted in the United States, four in the Netherlands, one in the UK, and the remaining three in Switzerland, Taiwan, and Italy. This information is presented in Table 1, which outlines the characteristics of the studies.

### 3.3. Risk of Bias in the Included Articles

**Figure 2:**
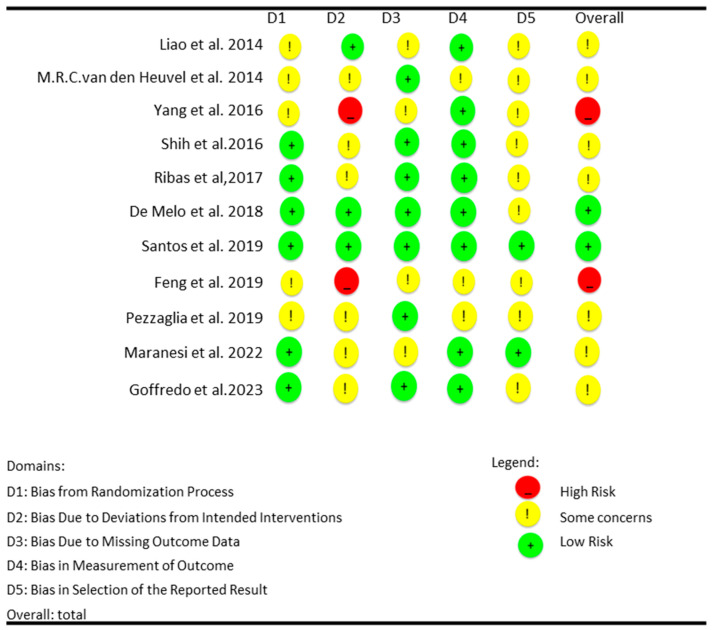
Cochrane risk-of-bias tool (RoB 2) [18,19,20,21,22,23,24,25,26,27,28].

The overall risk of bias varied among the included studies, with most showing some concerns, while a few exhibited high or low risk across specific domains. Issues with the randomization process were common, though several studies demonstrated a low risk. Deviations from the intended interventions posed a significant concern, particularly in Yang et al. (2016) [20] and Feng et al. (2019) [25], which were categorized as high risk. Most studies handled missing outcome data well, though some concerns were noted in specific cases. Outcome measurement biases were generally low, except for a few studies. Selection of the reported results raised concerns in multiple studies, except for Santos et al. (2019) [24] and Maranesi et al. (2022) [27], which had low risk. Overall, only Santos et al. (2019) [24] and De Melo et al. (2018) [23] had a consistently low risk of bias, while Yang et al. (2016) [20] and Feng et al. (2019) [25] were classified as high risk. These findings suggest that while some studies demonstrate strong methodological rigor, others require cautious interpretation due to potential biases affecting their outcomes [18,19,20,21,22,23,24,25,26,27,28] (Figure 2).

### 3.4. Sample Characteristics

Table 2 summarizes the findings of this systematic review, which included 518 participants diagnosed with Parkinson’s disease, with an average age of 67.3 years. Men comprised the majority of the sample (n = 352; 67.95%), while women accounted for 32.05% of the participants. The severity of Parkinson’s disease was assessed across all studies using the “Hoehn and Yahr stage” (H&Y) scale, yielding an average score of 2.03 out of 3. Notably, the study by Pazzaglia et al. (2019) [26] employed the “Unified Parkinson’s Disease Rating Scale” (UPDRS III), reporting a mean score of 24 out of 68 (Table 2).

As outlined in Table 3, all studies utilized virtual reality therapy as the primary intervention, though the implementation varied significantly. A notable difference was the integration of VR therapy with conventional physical therapy, which was applied in most studies. Exceptions to this were the studies by Yang et al. (2016) [20], De Melo et al. (2018) [23], Feng et al. (2019) [25], and Pazzaglia et al. (2019) [26]. The type of virtual reality tools used also differed between studies. Devices such as the “Kinect Xbox 360”, the Nintendo Wii Balance Board, and the “Tymo system” were among those employed. In the control groups, conventional therapy was the standard treatment, but its focus varied. Some studies emphasized balance training, while others implemented Proprioceptive Neuromuscular Facilitation (PNF) diagonals, treadmill exercises, therapeutic exercises, or muscle stretching.

In the majority of the studies included in this review (n = 6), the authors employed the Berg balance scale (BBS) to evaluate patients’ balance before and after the intervention. Alternatively, three studies opted for different assessment tools, such as the Tinetti Performance-Oriented Mobility Assessment (POMA), six-minute walking test (6 MWT), UPDRS III, and sensory organization test (SOT) score. The BBS, introduced in 1989, was specifically designed to assess both static and dynamic balance (Table 4). It consists of 14 tasks that assess various aspects of balance, such as sitting, standing, reaching, and turning. Each task is scored on a scale from 0 to 4, with higher scores indicating better balance and stability. The time required to complete the test is around 15–20 min. The BBS is commonly used in physical therapy and rehabilitation settings to monitor the progress of patients, particularly those with neurological or musculoskeletal conditions. The development of the BBS was introduced by Katherine Berg in 1989. Dr. Berg, a Canadian physiotherapist, designed the scale to provide a reliable and systematic measure of balance, particularly for older adults and individuals with conditions like stroke, Parkinson’s disease, and other neurological disorders. Her work has significantly contributed to the field of rehabilitation by offering a standardized method to assess balance and guide treatment interventions [29].

In the study conducted by Maranesi et al. (2022) [27], the POMA scale was used to evaluate balance and detect early fall risks. This tool includes nine items focused on balance and eight items dedicated to gait assessment. The POMA scale results from both sections are combined to determine the fall risk: a total score under 19 indicates a high risk, scores between 19 and 24 suggest a moderate risk, and scores between 25 and 28 represent a low risk of falls. In De Melo et al. (2018) [23], the six-minute walking test (6 MWT) was the physical assessment used, with the heart rate monitored before and after the intervention. This test evaluates aerobic capacity and endurance, with an increase in distance walked serving as an indicator of improved baseline mobility. This test is widely used in clinical settings to measure the functional exercise capacity [30].

The SOT used in Liao et al. (2014) [18] is an important balance assessment tool. It includes sensory tests to measure an individual’s ability to use visual, proprioceptive, and vestibular information to maintain postural stability in an upright position. The test is particularly useful for diagnosing balance disorders and assessing the effectiveness of treatments in conditions such as vestibular disorders, stroke, and other neurological conditions. The SOT is useful in determining whether balance issues are related to deficits in one of these sensory systems or due to difficulty integrating them effectively [31].

The Unified Parkinson’s Disease Rating Scale (UPDRS) is a clinical tool used to assess the severity of Parkinson’s disease (PD) in some studies. It consists of four sections: Part I—mental, behavioral, and mood aspects, Part II—activities of daily living (ADL), Part III—motor examination, and Part IV—motor complications. The UPDRS III specifically evaluates motor impairment through a series of clinician-administered tests. It includes assessments of the following: resting tremor, postural and kinetic tremor, muscle rigidity, bradykinesia (slowness of movement), postural instability, gait disturbances, and dyskinesias. Each item is scored on a 0 to 4 scale (0 = no impairment, 4 = severe impairment), with a total possible score ranging from 0 to 132. Higher scores indicate greater motor dysfunction. The UPDRS III is widely used in clinical practice and research to monitor the disease progression and treatment efficacy in Parkinson’s disease [32].

The results for “Balance” differed between the experimental and control groups. Significant improvements were noted in two studies: Feng et al. (2019) [25] and Maranesi et al. (2022) [27]. In the study by Feng et al. (2019) [25], the experimental group that received VR therapy showed a 14% improvement on the BBS compared to the control group, which received conventional therapy, with a 6% improvement. This indicates that the VR-based intervention provided a larger improvement in balance compared to traditional therapy, suggesting that VR may be more effective at enhancing balance in individuals with Parkinson’s disease.

In Maranesi et al. (2022) [27], combining virtual reality therapy with conventional therapy resulted in an 8% improvement in the POMA over five weeks. The control group, which received traditional rehabilitation, showed a 3% improvement. This also suggests that the VR-based intervention led to better outcomes in terms of balance and gait compared to the conventional therapy used in the study.

In De Melo et al. (2018) [23], the experimental group used the Kinect Xbox 360, while the control group performed treadmill training. Both treatments led to improvements in the 6 MWT, increasing both walking speed and distance. Other randomized controlled trials included in this review generally showed improvements in the experimental groups between pre- and post-intervention, regardless of whether they received virtual reality therapy alone or combined with conventional therapy.

The two trials that measured the results using the UPDRS III Motor were the study of Goffredo et al. (2023) and the study of van den Heuvel MR et al. (2014) [19,28]. Starting with the study by Goffredo et al. (2023), the experimental group participated in the intervention involving motor and cognitive rehabilitation exercises delivered through a non-immersive telerehabilitation (TR) approach based on virtual reality using the VRRS Tablet system (Khymeia Srl, Noventa Padovana, Padua, Italy) [19]. This system is a medical device authorized by the Italian Ministry of Health for neurological patient rehabilitation. Motor exercises were conducted with the aid of inertial sensors, which recorded and processed the patients’ movements. The treatments led to improvements in the UPDRS III, reducing the motor difficulties which can affect balance. The experimental group improved by 7%, while the control group, which received conventional treatment consisting of active exercise and stretching, improved by 0.74% on the UPDRS III. Regarding the percentage change between groups, the experimental group improved 18% more than the control group.

Table 4 displays the summary of the described results.

### 3.5. Quality of the Evidence

The evidence quality in this review is moderate regarding the evaluated variable, balance. The evaluation of the evidence quality largely focused on the risk of bias in the included clinical trials. A notable concern was the inadequate randomization process and the absence of blinding for both participants and personnel across all studies. Additionally, the small sample sizes, with most studies involving fewer than 400 participants, contributed to the overall imprecision of the findings (Table 5).

**Figure 3:**
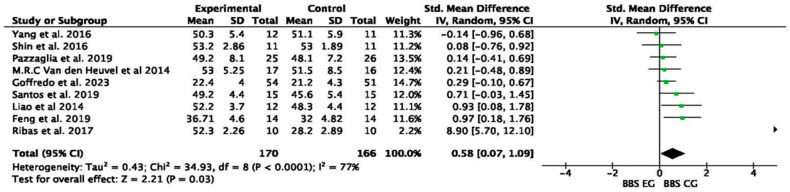
Forest plot of comparison: the BBS outcomes of experimental virtual reality group (EGs) versus traditional control groups (CGs) [18,19,20,21,22,24,25,26,28].

The most commonly used outcomes for assessing the effects of VR compared to conventional therapy were the BBS and 6 MWT. The forest plot of Figure 3 presents a meta-analysis comparing the BBS scores between experimental groups (BBS EGs) and control groups (BBS CGs) across 9 of the 11 studies. The overall standardized mean difference (SMD) is 0.58 (95% CI: 0.07 to 1.09), indicating a moderate effect size favoring the experimental groups. The test for the overall effect (Z = 2.21, *p* = 0.03) suggests a statistically significant difference between groups, indicating that the VR intervention had a positive impact on the balance performance. However, the heterogeneity is high (I^2^ = 77%), suggesting substantial variability among the studies. The confidence intervals for some individual studies cross zero, indicating inconsistent results, but the pooled effect remains significant.

**Figure 4:**
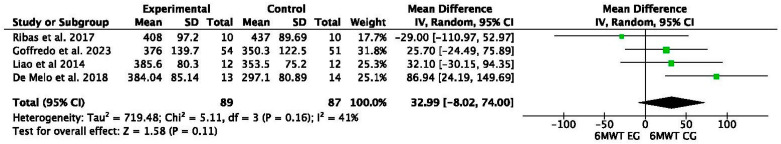
Forest plot of comparison: the 6 MWT outcomes of experimental virtual reality groups (EGs) versus traditional control groups (CGs) [18,22,23,28].

Figure 4 displays the comparison of the 6 MWT scores between the experimental groups (6 MWT EGs) and control groups (6 MWT CGs) across four studies. Overall, the pooled mean difference is 32.99 m (95% CI: −8.02 to 74.00), suggesting that the experimental groups walked more than the control groups, but the confidence interval includes zero, indicating no statistically significant difference. The test for the overall effect (Z = 1.58, *p* = 0.11) further confirms this lack of significance. The I^2^ value of 41% indicates moderate heterogeneity, meaning that while there is some variation among the included studies, it is not substantial. Additionally, the Chi^2^ value of 5.11 (*p* = 0.16) suggests that the observed differences across the studies are not statistically significant.

## 4. Discussion

In order to develop and respond to the study outcomes, this systematic review was carried out following the PRISMA 2020 version, where we obtained a total of 11 studies (randomized controlled clinical trials) for qualitative analysis. From the 11 studies included a total of 518 patients diagnosed with Parkinson’s were analyzed. In this review, 67.95% of participants were male and 32.04% were female, with a male-to-female ratio of approximately 2.12:1. Referring to the scientific evidence, in this pathology, the prevalence rate shows a slight tendency towards the male population [24]. Regarding the sample, the average age was 67.3 years, which aligns with the typical age of disease onset at 60 years [22,23]. Concretely, Ribas et al. (2018) report the youngest average age (60 years), De Melo et al. (2016) report an average of 62 years, and Maranesi et al. (2022), Yang et al. (2016), and Pazzaglia et al. (2019) report the oldest (72–75 years) [20,22,23,26,27]. Several studies report age means above 70 [20,26,27], while others fall in the mid-to-late 60s. Standard deviations vary significantly, ranging from 4.6 to 13.04 years, indicating substantial variation within some samples.

The virtual reality (VR) treatments explored in these studies included a variety of non-immersive and immersive exergaming approaches aimed at enhancing balance, motor coordination, and functional mobility. Several studies employed motion-tracking platforms, such as the Tymo^®^ system [27], Kinect sensors [21,23], and the Nintendo Wii Balance Board [18,22,24], to facilitate interactive exercises. In contrast, Pazzaglia et al. utilized a fully immersive VR rehabilitation system (NIRVANA), which integrates optoelectronic tracking for motion-based training [26]. Furthermore, Yang et al. and Van den Heuvel et al. introduced balance training using wireless boards and movement-tracking sensors [19,20], while Goffredo et al. adopted a telerehabilitation approach via the VRRS Tablet system [28]. Overall, these studies leveraged VR to promote motor function, postural control, and cognitive engagement through interactive virtual experiences.

As mentioned above, the scales used by the authors in the included studies were the BBS, POMA, 6 MWT, UPDRS III, and SOT score. The only minimal detectable change (MDC) found was by the Berg scale; for the other scales used, it was not possible to find this value for Parkinson’s patients. The minimum detectable change by the BBS for patients with Parkinson’s was 5 points [30].

A meta-analysis of 48 studies evaluated the reliability of the BBS. The internal consistency was measured in 19 studies (Cronbach’s alpha = 0.897), the inter-rater reliability in 29 studies (ICC = 0.939), and the intra-rater reliability in 30 studies (ICC = 0.937), using a fixed-effects model. The MDC was determined by analyzing the reliability variability, incorporating standard deviation and reliability coefficients. The variability among the studies suggests that the BBS reliability may not be universally applicable across all contexts [33].

For the POMA, a study involving older adults residing in long-term care facilities reported MDCs of 4.2 and 4.0 for individual assessments by two raters, and 0.8 and 0.7 for group assessments by the same raters [34]. Regarding the 6 MWT, research focusing on PD found an MDC of 82 m, indicating that changes less than this may not reflect a true change in walking capacity [35]. For the SOT, specific MDC values are not available in the literature.

It is important to note that MDC values can vary based on the population studied and the specific methodologies employed. Therefore, when interpreting changes in these assessments, it is crucial to consider the context and the specific characteristics of the population being evaluated.

In our systematic review, the analyzed studies showed results with significant improvements in the balance variable, especially in the studies by Liao et al. (2014) [18] and Feng et al. (2019) [25].

Concretely, in Liao et al. (2014), the experimental group showed a 15% improvement in the SOT, and the control group, which received conventional physical therapy, demonstrated only a 5% improvement [18].

In Feng et al. (2019), the experimental group experienced a 14% improvement on the BBS. The control group, which received conventional therapy, showed a 6% improvement. Although the experimental group showed a larger improvement, it is important to know whether the improvement in the BBS was statistically significant [25].

Both studies suggest favorable outcomes for VR interventions, but clearer details on the statistical significance of the improvements and the control groups’ performances would strengthen the evidence supporting VR’s efficacy in these contexts.

In the randomized controlled clinical trial of Laio et al. (2014) [18], in the experimental group, the intervention was VR treatment, treadmill, and conventional exercise such as stretching and muscle strengthening. The combination of the aforementioned treatments, in the same session, obtained 15% more improvement than the control group, along with 16% more improvement pre- and post-intervention. In the trial of Feng et al. (2019) [25], the experimental group underwent balance training with VR. The patients were in front of a large screen performing active-game-type exercises to develop a certain function using the upper and lower extremities and, at the same time or independently, to actively respond to the requested game function, such as hitting a ball or training walking at different rhythms determined by the game. All these active and fun activities provided to the experimental group for 12 weeks obtained an improvement of 14% more than the control group on the BBS, matching the minimum detectable change on the same scale, indicative of the minimum cause of change that is not due to the measurement tool. So, according to this study, VR is a promising treatment that can provide benefits in balance treatments by directly increasing the patient’s ability to care for themselves and reducing the burden on caregivers [25]. In the study by De Melo et al. (2014) [23], in the experimental group, participants were placed at a distance of 3 m in front of a Kinect Xbox 360TM motor sensor, which is also a projector that projected the treatment activities. Thanks to the various simulations of activities, the experimental group together with the control group that worked with the treadmill obtained the greatest distance in the 6 MWT and the highest speed post-6 MWT compared to the control group [23]. Referring to both studies, the VR interventions were standardized across participants to maintain consistency and reliability in assessing the outcomes [23,25].

In 9 of the 11 included studies, the severity of the sample was assessed using the H&Y scale, which represents a classification by stages of easy application and is related to the motor deterioration and quality of life of the patient with Parkinson’s, reflecting a mild–moderate affectation of the population examined by these studies.

Mild cases (Stage 1–2) were reported in De Melo et al. (2018) [23] (H&Y = 1.45–2.08) and Santos et al. (2019) [24] (H&Y = 1.3–1.5). Moderate cases (Stage 2) were observed in Liao et al. (2014) [18] (H&Y = 1.9–2) and Maranesi et al. (2022) [27] (H&Y = 2). Advanced moderate cases (Stage 3) were noted in Yang et al. (2016) [20] (H&Y = 3). Pazzaglia et al. (2019) [26] assessed severity using the UPDRS instead of the H&Y.

The UPDRS was also used in the study by van den Heuvel MR (2014) [19] and Goffredo et al. (2023) [28]. The populations of both studies obtained scores between 23 and 25 out of 68, indicating a moderate affectation of the motor symptoms of Parkinson’s disease.

Balance and gait are predictive factors of the future conditions of the independence and functionality of patients with Parkinson’s, associated with the risk of falling, walking, and mental capacity above all; these deficits mark difficulties in daily life [36].

This fact is supported by the findings in Lichter et al. (2018), which emphasize that gait and balance disorders are crucial risk factors not only for mobility issues but also for cognitive decline, dementia, and other non-motor symptoms in individuals with Parkinson’s disease. Their research highlights the significant role that motor symptoms, particularly balance and gait disturbances, play in predicting functional independence and overall quality of life for PD [37].

VR represents the main intervention of our study and can mediate these problems. In patients with Parkinson’s, there is often an inadequate interaction between sensory systems such as the vestibular, visual, and somatosensory proprioceptive systems. VR, with its ability to generate a safe and immersive environment, with various “feedbacks”, increases brain neuroplastic activation and also the patient’s level of cognitive attention, generating a fun experience. Another great benefit of this treatment is its versatility and its ability to be perfectly integrated with another treatment, of a conventional type, which can further increase the physiological effects previously explained [38]. The clinical trials of Bacha et al. (2021) [36] and Cikajlo et al. (2019) [39] indicate how rehabilitation with VR is evolving rapidly and has great power, also supplied independently or coupled with other therapies, in the treatment of balance and gait in patients with Parkinson’s [35]. Another highly relevant aspect is the safety of this therapy, which can generate a safe environment where the patient can experience their motor experience without any risk. In this aspect, we find consistency with the results of the study by Kim et al. (2017) [40] and the existing scientific literature. In addition, several other research articles further support the safety of virtual reality (VR) therapy for individuals with Parkinson’s disease. Canning et al. (2020) reviewed the use of VR in gait and balance rehabilitation for Parkinson’s patients, noting that VR interventions are generally well tolerated and free of significant safety concerns [2]. They found that VR therapy can improve motor outcomes without posing additional risks. Similarly, Lei et al. (2019) conducted a systematic review and reported that VR rehabilitation training was safe for Parkinson’s patients, with no major adverse effects observed [41]. Furthermore, Triegaardt et al. (2020) performed a meta-analysis of 1031 participants and concluded that VR therapy is not only effective at improving motor function but is also safe, with no significant adverse events reported in the majority of the studies reviewed [42]. Collectively, these studies highlight the safety of VR therapy as a promising tool for rehabilitation in Parkinson’s disease [38,39,40].

## 5. Conclusions

This systematic review highlights the potential of VR-based interventions in enhancing balance outcomes among individuals with PD. By synthesizing evidence from RCTs, the following key findings were identified:

1. VR-based therapies were found to be as effective, if not superior, to conventional therapies at improving balance-related outcomes, with significant gains observed in dynamic balance, postural stability, and fall risk reduction;
2. The combination of VR with conventional therapy demonstrated added benefits compared to standalone interventions, suggesting that an integrated approach may provide enhanced outcomes through the complementary strengths of both methods.

In conclusion, VR-based interventions hold significant promise as a novel therapeutic tool for addressing balance impairments in PD, particularly when tailored to individual needs and integrated with conventional therapies. Future research should focus on optimizing intervention protocols, exploring cost effectiveness, and evaluating long-term impacts to facilitate wider clinical adoption.

### Recommendations

VR-based balance training is an emerging intervention for PD that enhances postural control and reduces the fall risk. Effective VR therapy should incorporate task-oriented training, focusing on weight shifting, stepping strategies, and postural adjustments. Dual-task training, integrating cognitive and motor tasks, improves real-world applicability, while exergaming-based therapy enhances engagement. Semi-immersive and non-immersive VR systems are preferred to reduce motion sickness and fatigue.

For optimal outcomes, VR training sessions should last 30–60 min, be conducted three–five times per week, and continue for a minimum of 6–12 weeks to achieve meaningful balance improvements. Longer interventions may provide sustained benefits.

Key VR system features include real-time feedback (auditory, visual, or haptic) to support motor learning, adaptive difficulty to ensure progressive challenge, and motion tracking using sensors for accurate postural assessment. Multi-sensory stimulation (visual, auditory, and proprioceptive) further enhances sensorimotor integration. Safety measures, such as harness support or therapist supervision, are essential for patients with advanced PD.

Long-term adherence can be improved through home-based VR therapy using user-friendly and portable solutions. Integrating VR with conventional physical therapy (e.g., strength training, gait exercises) enhances the overall effectiveness. To maintain balance gains, weekly maintenance sessions (one–two times per week) are recommended post-intervention.

These recommendations, based on research and clinical best practices, highlight the importance of individualized therapy design, considering the disease progression and patient-specific needs.

## Figures and Tables

**Table 1:** Characteristics of studies.

Author	Year	Design	Journal	Country
Liao et al. [18]	2014	RCT	Neurorehabilitation and Neural Repair	USA
M.R.C. van den Heuvel et al. [19]	2014	RCT	Elsevier	NETHERLANDS
Yang et al. [20]	2016	RCT	Journal of the Formosan Medical Association	TAIWAN
Shin et al. [21]	2016	RCT	Journal of NeuroEngineering and Rehabilitation	UK
Ribas et al. [22]	2017	RCT	Elsevier	NETHERLANDS
De Melo et al. [23]	2018	RCT	NeuroRehabilitation	NETHERLANDS
Santos et al. [24]	2019	RCT	NeuroRehabilitation	NETHERLANDS
Feng et al. [25]	2019	RCT	Medical Science Monitor	USA
Pazzaglia et al. [26]	2019	RCT	Elsevier	USA
Maranesi et al. [27]	2022	RCT	International Journal of Environmental Research and Public Health	SUISA
Goffredo et al. [28]	2023	RCT	European Journal of Physical and Rehabilitation Medicine	ITALY

Legend: RCT—randomized controlled trial; USA—United States of America.

**Table 2:** Characteristics of the analyzed sample.

Author	Year	Design	Size	Years	Gender	Severity
Liao et al. [18]	2014	RCT	EG pre: 12/post: 12	67 (7.1)	6 M 6 F	H&Y = 2 (0.7)
			CG: pre: 12/post: 11	64 (8.6)	5 M 7 F	H&Y = 1.9 (0.8)
			TE: pre: 12/post: 12	65 (6.7)	6 M 6 F	H&Y = 2 (0.8)
M.R.C.van den Heuvel et al. [19]	2014	RCT	EG: pre: 17/post: 17	66.3 (6.39)	12 M 5 F	H&Y = 2.5
			CG: pre: 16/post: 14	68.8 (9.68)	8 M 8 F	H&T = 2.5
Yang et al. [20]	2016	RCT	EG: pre: 12/post: 10	72.5 (8.4)	7 M 4 F	H&Y = 3 (3.3)
			CG: pre: 11/post: 10	75.4 (6.3)	7 M 5 F	H&Y = 3 (3.3)
Shih et al. [21]	2016	RCT	EG: pre: 11/post: 10	67.5(9.9)	9 M 1 F	H&Y = 1.6 (0.8)
			CG: pre: 11/post: 10	68.8 (9.6)	7 M 3 F	H&Y = 1.4 (0.52)
Ribas et al. [22]	2017	RCT	EG: pre: 10/post: 10	61.70 (6.8)	4 M 6 F	H&Y = 1.25
			CG: pre: 10/post: 10	60.20 (11.2)	4 M 6 F	H&Y = 1.5
De Melo et al. [23]	2018	RCT	EG2: pre: 13/post: 12	60 (9.28)	11 M 1 F	H&Y = 1.45 (0.51)
			CG: pre: 14/post: 12	65 (13.04)	5 M 7 F	H&Y = 2.08 (0.9)
			EG1: pre: 15/post: 13	61 (10.72)	12 M 1 F	H&Y = 1.53 (0.66)
Santos et al. [24]	2019	RCT	EG: pre: 15/post: 14	66.6 (8.2)	9 M 5 F	H&Y = 1.5 (0.4)
			EG1: pre: 15/post: 13	61.7 (7.3)	11 M 2 F	H&Y = 1.4 (0.6)
			GC2: pre: 15/post: 14	64.5 (9.8)	11 M 3 F	H&Y = 1.3 (0.3)
Feng et al. [25]	2019	RCT	EG: pre: 14/post: 14	67 (4.7)	8 M 7 F	H&Y = 3.03 (0.55)
			GC: pre: 14/post: 14	66 (4.6)	9 M 6 F	H&Y = 2.97 (0.58)
Pazzaglia et al. [26]	2019	RCT	EG: pre: 25/post: 25	72 (7)	18 M 7 F	UPDRS III = 23 (9)
			GC: pre: 26/post: 26	70 (10)	17 M 9 F	UPDRS III = 25 (10)
Maranesi et al. [27]	2022	RCT	EG: pre: 16/post: 16	72 (6.3)	6 M 10 F	H&Y = 2
			GC: pre: 16/post: 14	75 (5.4)	9 M 5 F	H&Y = 2
Goffredo at al. [28]	2023	RCT	EG: pre: 54/post: 49	67.8 (6.6)	27 M 22 F	H&Y = 2
			GC: pre: 51/post: 48	68.2 (5.8)	24 M 24 F	H&Y = 2

Legend: H&Y—Hoehn and Yahr stage; UPDRS—Unified Parkinson’s Disease Rating Scale; EG—experimental group; CG—control group; RCT—randomized controlled trial; M—males; F—females.

**Table 3:** Characteristics of all the interventions analyzed.

Author	Year	Design	Intervention Characteristics	Intensity	Reps/Series	Frequency	Session Duration	Intervention Duration
Liao et al. [18]	2014	RCT	Fall prevention education	n/m	10–15/3	2 sessions/week	60 min	6 weeks
			Traditional exercise + treadmill	n/m	10–15/3	2 sessions/week	60 min	6 weeks
			Wii Fit VR + traditional exercise + treadmill	n/m	10–15/3	2 sessions/week	60 min	6 weeks
M.R.C. van den Heuvel et al. [19]	2014	RCT	VR balance training Conventional balance training	n/m n/m	n/m n/m	2 sessions/week 2 sessions/week	60 min 60 min	5 weeks 5 weeks
Shin et al. [20]	2016		Kinect sensor, Microsoft Conventional balance training	n/m n/m	n/m n/m	2 sessions/week 2 sessions/week	50 min 50 min	8 weeks 8 weeks
Yang et al. [21]	2016	RCT	VR balance training	n/m	3 × 10 min	2 sessions/week	50 min	6 weeks
			Conventional balance training	n/m	3 × 10 min	2 sessions/week	50 min	6 weeks
Ribas et al. [22]	2017	RCT	Wii Fit games, Nintendo	n/m	n/m	2 sessions/week	30 min	12 weeks
			Warming + stretching and active exercises + resistance exercise and diagonal exercise for the trunk, neck, and limbs	n/m	n/m	2 sessions/week	30 min	12 weeks
De Melo et al. [23]	2018	RCT	Kinect Xbox 360^TM^	60–70% HB	n/m	3 sessions/week	20 min	4 weeks
			Traditional exercise	60–70% HB	n/m	3 sessions/week	20 min	4 weeks
			Treadmill	60–70% HB	n/m	3 sessions/week	20 min	4 weeks
Santos et al. [24]	2019	RCT	Nintendo + Wii Balance Board platform + FNP	n/m	n/m	2 sessions/week	50 min	8 weeks
			Nintendo + Wii Balance Board platform + FNP	n/m	n/m	2 sessions/week	50 min	8 weeks
			FNP diagonals	n/m	n/m	2 sessions/week	50 min	8 weeks
Feng et al. [25]	2019	RCT	RV	n/m	n/m	5 sessions/week	45 min	12 weeks
			Traditional exercise	n/m	n/m	5 sessions/week	45 min	12 weeks
Pazzaglia et al. [26]	2019	RCT	VR session with multiple exercises	n/m	n/m	3 sessions/week	40 min	6 weeks
			Traditional exercise	n/m	n/m	3 sessions/week	40 min	6 weeks
Maranesi et al. [27]	2022	RCT	Traditional exercise + Tymo system	n/m	n/m	2 sessions/week	50 min	5 weeks
			Traditional exercise	n/m	n/m	2 sessions/week	50 min	5 weeks
Goffredo et al. [28]	2023	RCT	VRRS Tablet	n/m	n/m	3–5 sessions/week	45 min	6–10 weeks
			Stretching + active exercise	n/m	10/1	3–5 sessions/week	45 min	6–10 weeks

Legend: HB—heartbeat; n/m—not mentioned; FNP—proprioceptive neuromuscular facilitation; VR—virtual reality; traditional exercise—warm-up + coordination + balance + physical evaluation characteristics.

**Table 4:** Results of the analyzed studies.

Author	Year	Design	Equilibrium Outcomes
Liao et al. [18]	2014	RCT	EG pre–post: 16% improvement in SOT score
			CG: no significant changes
			TE pre–post: 9% improvement in SOT score
			EG vs. CG: 15% more improvement than CG
			EG vs. TE: 2% more improvement than TE
M.R.C.van den Heuvel et al. [19]	2014		EG pre–post: 96.43% reduction on UPDRS III MOTOR
			CG pre–post: 85.4% reduction on UPDRS III MOTOR EG vs. CG: −11.03%
Yang et al. [20]	2016	RCT	EG pre–post: 3 pt (7%) improvement on BBS
			CG pre–post: 3 pt (6%) improvement on BBS
			EG vs. CG: no differences on BBS
Shin et al. [21]	2016		EG pre–post: 2.3 pt (4.5%) improvement on BBS CG pre–post: 2.6 pt (5.1%) improvement on BSS EG vs. CG: 0.37% more improvement than GC
Ribas et al. [22]	2017		EG pre–post: 1.9 pt (3.7%) improvement on BBS CG pre–post: 0.20 pt (0.41%) improvement on BBS EG vs. CG: 8.5% more improvement than CG
De Melo et al. [23]	2018	RCT	EG pre–post: 6% improvement in 6 MWT HR
			CG pre–post: no significant changes
			CG 2 pre–post: 6% improvement in 6 MWT HR
			EG vs. CG2: 3% more improvement than CG2
Santos et al. [24]	2019	RCT	EG pre–post: 5.5 pt (13%) improvement on BBS
			CG 1 pre–post: 5 pt (13%) improvement on BBS
			CG 2 pre–post: 5 pt (12%) improvement on BBS
			EG vs. CG 1: no differences on BBS
			EG vs. CG 2: no differences on BBS
Feng et al. [25]	2019	RCT	EG pre–post: 6 pt (19%) improvement on BBS
			CG pre–post: 2 pt (6%) improvement on BBS
			EG vs. CG: 14% more improvement than GC
Pazzaglia et al. [26]	2019	RCT	EG pre–post: 5 pt (20%) improvement on BBS
			CG pre–post: 4 pt (17%) improvement on BBS
			EG vs. CG: no differences on BBS
Maranesi et al. [27]	2022	RCT	EG pre–post: 6% improvement in POMA balance
			GC pre–post: 8% improvement in POMA balance
			EG vs. CG: 8% more improvement than CG
Goffredo et al. [28]	2023		EG pre–post: 2.6 pt (7%) improvement on UPDRS III MOTOR CG pre–post: 0.3 pt (0.74%) improvement on UPDRS III MOTOR EG vs. CG: 18% more improvement than CG

Legend: EG—experimental group; CG—control group; TE—traditional exercise; BBS—Berg balance scale; SOT—sensory organization test score; 6 MWT—six-minute walking test; POMA—Tinetti Performance-Oriented Mobility Assessment.

**Table 5:** GRADE evidence quality. Question: virtual reality therapy compared to conventional physiotherapy for Parkinson’s disease. Setting: physiothearapy clinics.

Certainty Assessment							Impact		
No. of Studies	Study Design	Risk of Bias	Inconsistency	Indirect Evidence	Imprecision	Other Considerations		Certainty	Importance
Balance Follow-Up: Range: 4 Weeks; Assessed with Various Scales (BBS, SOT Score, Tinetti POMA, 6 MWT HR)									
11	Randomized trial	Very serious	Not serious	Not serious	Extremely serious	Dose-response gradient	Two studies showed improvements in balance of 14% and 15% in favor of the virtual reality therapy group. Three studies did not show significant improvements, while two studies showed improvements of 8% and 3% in favor of the experimental group, the virtual reality group.	⨁◯◯◯ Very low	Critical

Explanations: A serious risk of bias due to the lack of proper randomization in many studies, as well as the lack of blinding of both participants and personnel in all studies.

## Data Availability

The data can be accessed upon submitting a reasonable request to the corresponding author.
